# Antiretroviral Drugs Impact Autophagy with Toxic Outcomes

**DOI:** 10.3390/cells10040909

**Published:** 2021-04-15

**Authors:** Laura Cheney, John M. Barbaro, Joan W. Berman

**Affiliations:** 1 Division of Infectious Diseases, Department of Medicine, Montefiore Medical Center and Albert Einstein College of Medicine, 1300 Morris Park Ave, Bronx, NY 10461, USA; 2 Department of Pathology, Montefiore Medical Center and Albert Einstein College of Medicine, 1300 Morris Park Ave, Bronx, NY 10461, USA; john.barbaro@einsteinmed.org (J.M.B.); joan.berman@einsteinmed.org (J.W.B.); 3 Department of Microbiology and Immunology, Montefiore Medical Center and Albert Einstein College of Medicine, 1300 Morris Park Ave, Bronx, NY 10461, USA

**Keywords:** HIV, antiretroviral drugs, side effects, toxicity, autophagy, mitophagy, mitochondria, ER stress

## Abstract

Antiretroviral drugs have dramatically improved the morbidity and mortality of people living with HIV (PLWH). While current antiretroviral therapy (ART) regimens are generally well-tolerated, risks for side effects and toxicity remain as PLWH must take life-long medications. Antiretroviral drugs impact autophagy, an intracellular proteolytic process that eliminates debris and foreign material, provides nutrients for metabolism, and performs quality control to maintain cell homeostasis. Toxicity and adverse events associated with antiretrovirals may be due, in part, to their impacts on autophagy. A more complete understanding of the effects on autophagy is essential for developing antiretroviral drugs with decreased off target effects, meaning those unrelated to viral suppression, to minimize toxicity for PLWH. This review summarizes the findings and highlights the gaps in our knowledge of the impacts of antiretroviral drugs on autophagy.

## 1. Introduction

### 1.1. Antiretroviral Therapy

Almost 33 million people worldwide have died since the beginning of the human immunodeficiency virus (HIV) epidemic that began nearly 40 years ago, and approximately 38 million people worldwide are currently living with HIV [1]. It is one of the most impactful epidemics in human history, with far reaching social, economic, and political ramifications as well.

**Figure 1:**
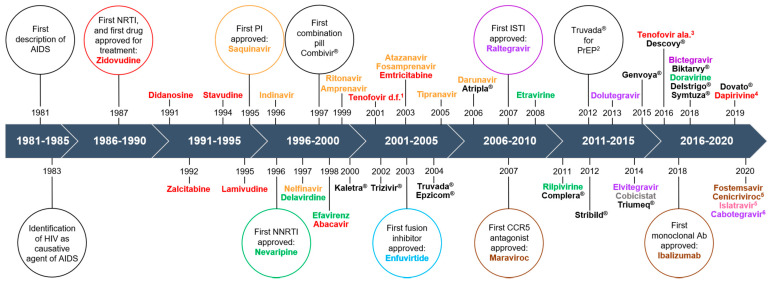
Timeline of antiretroviral drug development. Major milestones are encircled. Individual drugs are shown with the year the United States Food and Drug Administration granted approval for use. Individual drug names are colored coded by drug class: red = nucleoside reverse transcriptase inhibitor (NRTI); orange = protease Inhibitor (PI); green = non-nucleoside reverse transcriptase inhibitor (NNRTI); blue = fusion inhibitor; purple = integrase strand transfer inhibitor (ISTI); brown = entry inhibitor. Islatravir, in pink, is the first nucleoside reverse transcriptase translocation inhibitor (NRTTI). Cobicistat, in gray, is an analogue of ritonavir, and the first antiretroviral booster that does not have antiviral activity. Registered brand names, in black, are used for combination pills for space considerations. ^1^ tenofovir disoproxil fumarate; ^2^ PrEP = pre-exposure prophylaxis; ^3^ tenofovir alafenamide; ^4^ FDA approval pending for vaginal ring formulation; ^5^ currently in phase III trials; ^6^ approved in early 2021 as a long-acting injectable, the first of its kind. It is co-administered with a long-acting formulation of rilpivirine.

**Figure 2:**
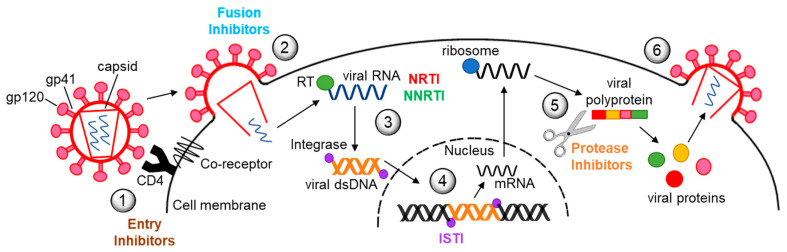
The HIV life cycle and antiretroviral drug targets. (**1**) The HIV genome consists of two positive-sense single strand RNA molecules, enclosed by a capsid. The capsid is surrounded by a lipid bilayer envelope which is studded with the viral transmembrane glycoprotein gp41, with viral gp120 positioned on top. Gp120 binds to the cluster of differentiation 4 (CD4) receptor, inducing a conformational change that enables it to then bind to either of two co-receptors on the cell surface, C-C chemokine receptor type 5 (CCR5) or C-X-C chemokine receptor type 4 (CXCR4). (**2**) After binding to a coreceptor, the viral envelope fuses with the cell membrane, followed by release of the capsid, genome, and viral proteins into the cytosol. (**3**) The RNA genome is reverse transcribed in the cytosol by reverse transcriptase (RT) into linear double-stranded DNA, which is then imported into the nucleus where it (**4**) integrates into the cell genome by the action of Integrase. Cell machinery transcribes HIV proviral DNA into single-stranded mRNA that is exported out of the nucleus into the cytoplasm for translation. (**5**) Viral assembly begins when HIV protease cleaves viral polyproteins into individual functional proteins. (**6**) Viral proteins and the genome are packaged into newly assembled virions that bud from the cell surface, incorporating the lipid bilayer of the host cell membrane. The color coding for drug classes established in Figure 1 is maintained here, and class names are positioned in the figure where they act within the life cycle. To date, there are no medications that block viral budding. NRTI = nucleoside reverse transcriptase inhibitor; NNRTI = non-nucleoside reverse transcriptase inhibitor; ISTI = integrase strand transfer inhibitor.

Decades of research have led to the development of more than 30 different antiretroviral drugs for treatment of HIV infection (Figure 1). Currently, there are six major classes of antiretrovirals, each targeting a different step in the virus life cycle (Figure 2). Antiretroviral therapy (ART) has dramatically reduced mortality for people living with HIV (PLWH). As a result of expanding access and changes in treatment guidelines, approximately 67% of PLWH worldwide are currently taking ART [1]. Additionally, at the end of 2020, over 900,000 people in 69 countries were receiving antiretroviral drugs to prevent HIV infection, called pre-exposure prophylaxis (PrEP) [2].

Historically, ART regimens consisted of high pill burdens, difficult dosing schedules, and toxicities that rendered compliance difficult. Toxicities are broad, including lactic acidosis, metabolic syndrome and diabetes, lipodystrophy, gastrointestinal distress, hepatotoxicity, pancreatitis, nephrotoxicity, increased cardiovascular risks, hypersensitivity reactions, cutaneous reactions, and neuropsychiatric disorders. As ART regimens have evolved, the quality of life for PLWH has also dramatically improved with significantly reduced pill burdens and substantially decreased side effects. No single antiretroviral drug nor drug combination can eliminate the virus from the body, thus necessitating life-long treatment. Therefore, long-term toxicity remains a concern for PLWH, and even relatively new antiretroviral drugs have burdensome side effects [3,4,5].

Off target effects of antiretroviral drugs, occurring either directly or indirectly on mitochondria and endoplasmic reticulum (ER), contribute to side effects, and have been studied extensively. Mitochondrial damage and ER stress modulate autophagy, a vital cell process necessary for cellular homeostasis. However, antiretroviral drugs also impact autophagy independently from mitochondrial damage and ER stress. Yet there is limited understanding of the effects of antiretroviral drugs on autophagy and the resulting toxicity and side effects (Table 1). This is the focus of our review.

**Table 1:** Overview of reviewed studies.

Antiretroviral Drug	Cell Type/Animal Model	Disease Process	Cell Toxicity/Effect	Autophagy Effect ^1,^*	Ref.
Rev. Transc. Inhibitors					
Efavirenz	SH-SY5Y, primary rat neurons	Neurotoxicity, HAND	Mitochondrial, apoptosis	Induced autophagy/mitophagy	[6]
	Hep3B, Hela	Hepatoxicity	Mitochondrial	*Dose-dependent auto/mitophagy inhibition	[7]
	Hep3B, primary rat neurons	Hepato-, neurotoxicity	ROS, mitochondrial, ER stress, cell death	Increased LC3-II	[8]
	primary human keratinocytes	Cutaneous reactions	Terminal differentiation, cell death	Decreased phospho-mTOR, increased LC3-lI	[9]
	EA.hy926, HUVEC	Cardiac, endothelial toxicity	ER stress, decreased meshwork, viability	Increased number of APG	[10]
	hCMEC/D3, human BMVEC, Tg HIV mice	Neurotoxicity, HAND	ER stress	* Inhibited autophagy	[11]
	U251-MG	Neurotoxicity, HAND	Mitochondrial	Inhibited autophagy/mitophagy	[12]
Zidovudine	C2C12	Myopathy	ROS, mitochondrial, cell viability	* Inhibited autophagy	[13]
	293T, 3T3-F442A	Lipoatrophy	ROS, mitochondrial, cell death	* Inhibited autophagy	[14]
	HepG2, HUH7	Hepatotoxicity	ROS, mitochondrial, apoptosis	* Inhibited autophagy	[15]
	male Wister rats, hepatocytes	Hepatocarcinogenesis	Mitochondrial	Initiation inhibition, maturation inhibition	[16]
	Primary Sprag.-Dawl. rat oocytes	Low fertility	Decreased maturity/cleavage, apoptosis	Increased autophagy	[17]
	Primary human PBMC	Immunologic recovery	ROS, mitochondrial, apoptosis	* No change in autophagy activity factor	[18]
	HUVEC, human aortic endothelial cells	Cardiac, endothelial toxicity	Mitochondrial	* Increased LC3-II, mito.: lysosome co-local.	[19]
Stavudine	293T, 3T3-F442A	Lipodystrophy	ROS, mitochondrial, apoptosis	* Inhibited autophagy	[14]
	HepG2, HUH7	Hepatoxicity	ROS, mitochondrial, cell death	* Inhibited autophagy	[15]
Lamivudine	Primary Sprag.-Dawl. rat oocytes	Low fertility	Decreased maturity/cleavage, apoptosis	Increased autophagy	[17]
	HUVEC, human aortic endothelial cells	Cardiac, endothelial toxicity	ROS, mitochondrial, apoptosis	* Increased LC3-II, mito.:lysosome co-local.	[19]
Protease Inhibitors					
Lopinavir/Ritonavir	3T3-L1, human SGBS adipocytes	Lipodystrophy	ER stress, inhibited differentiation, apoptosis	* Inhibited autophagy	[20]
	Primary mouse hepatocytes	Hepatoxicity	ER stress, dec. ROS response, cell death	Increased LC3-II	[21]
	Human JEG3, 3A-subE cells	Placenta health	ER stress	* Increased number of APG	[22]
Atazanavir	Human JEG3	Placenta health	ER stress	Increased number of APG	[22]
	SE872	Lipodystrophy	Decreased lipid stores, differentiation	Increased autophagy/mitophagy	[23]
Saquinavir	Chub-S7	Lipodystrophy	ROS, mitochondrial, apoptosis	* Increased autophagy genes mRNA, APG	[24]
Combinations					
TDF + FTC + DTG^2^	Primary Sprag.-Dawl. rat microglia	HAND	Increased mRNA for inflammatory markers	Inhibited autophagy, lysosome dysfunction	[25]
TDF + FTC + DTG	Primary Sprag.-Dawl. rat microglia	HAND	ROS	Inhibited autophagy, lysosome dysfunction	[26]
TEN + FTC + RAL ^3^	Primary human astrocytes	HAND	Effects aside from autophagy not assessed	* Inhibited autophagy	[27]
ZDV + SQV + NVP + Intlnh ^4^	Primary Sprag.-Dawl. rat neurons	HAND	Decreased neuron health markers, ATP	Increased autophagy	[28]
FTC + RTV + ATV ^5^	HIV infected primary human astrocytes	HAND	Increased viral and cytokine production	Increased p62	[29]
2 or 3-drug regimens ^6^	Primary Human PBMC	Immune senescence	Increased pro- & anti-apoptotic gene mRNA	* Decreased *BECN1*, increased *LC3* mRNA	[30]

^1^ Major findings are reported when flux or multiple autophagy assays were not performed. * Studies that used clinically therapeutic concentrations of antiretroviral drugs. ^2^ tenofovir disoproxil fumarate + emtricitabine + dolutegravir. ^3^ tenofovir + emtricitabine + raltegravir. ^4^ zidovudine + saquinavir + nevirapine + integrase inhibitor. ^5^ emtricitabine + ritonavir + atazanavir. ^6^ drug classes, but not individual drugs, were defined.

### 1.2. Autophagy

Autophagy is a conserved catabolic process in which intracellular substrates, including macromolecules, organelles, protein aggregates, and infectious agents, are degraded inside lysosomes. The most well-characterized purpose of autophagy is to protect cells from starvation and related stress. It also regulates multiple cell processes that contribute to cellular, organ, and organism homeostasis. Autophagy, or dysregulation of autophagy, contributes to physiological aging and to the pathogenesis of many diseases, including hepatic and cardiac diseases, myopathies, cancer, infections, and neurodegeneration.

**Figure 3:**
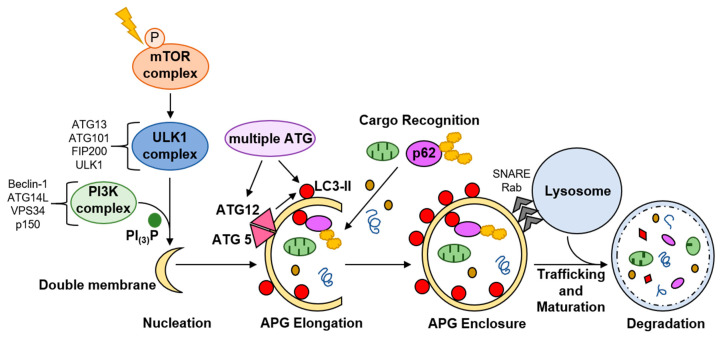
Simplified schematic of macroautophagy. There are more than 30 proteins directly involved in this highly coordinated process. A stimulus induces phosphorylation of mTOR, inactivating the mTOR complex. This releases the UNC-51 like kinase (ULK1) pre-initiation complex from inhibition, which then recruits a class III phosphatidylinositol 3-kinase (PI3K) complex. This enriches for phosphatidylinositol-3-phosphate (PI_(3)_P) at intracellular membrane sites, most commonly the ER and mitochondria, resulting in nascent double membrane formation. This new autophagosome (APG) is docked by a functional autophagy-related (ATG) 5–ATG12 complex that is activated by other ATG proteins. This complex, in concert with other ATG proteins, processes LC3: LC3 is cleaved and lipidated with phosphatidylethanolamine to form LC3-II that associates with the inner and outer membrane of the forming APG to facilitate elongation and enclosure as well as cargo recognition. Sequestrome-1 (SQSTM1/p62), one autophagy receptor, binds to LC3-II to include specific cargo inside the forming APG, imparting selectivity to degradation. The APG membrane closes around cargo, is trafficked to a lysosome, and its outer membrane fuses with a lysosome through the action of Rab family GTPases, and soluble N-ethylmaleimide-sensitive factor-attachment protein receptor (SNARE) superfamily proteins in a process called maturation. This results in creation of the autolysosome and degradation of APG cargo. While the LC3-II on the inner APG membrane is degraded within the autolysosome, the LC3-II on the outer membrane can be recycled back to LC3-I to participate again in APG biogenesis.

Macroautophagy, hereafter called autophagy, is the most well-characterized form of autophagy. It involves formation of a double membrane vesicle, termed an autophagosome (APG), around substrates to be degraded, which then fuses, most commonly, with a lysosome (Figure 3). Microautophagy and chaperone-mediated autophagy are two other major types of autophagy in which cargo enters directly into the lysosome. These will not be discussed.

Autophagy is highly regulated and dynamic, responsive to various signals and stressors. Canonical autophagy is initiated when mammalian target of rapamycin (mTOR) mTOR is inactivated by phosphorylation. A cascade of phosphorylation events, phosphatidylinositol signaling, and protein complex recruitment culminates in formation of a nascent double membrane [31] (Figure 3). The nascent APG is elongated, cargo is enclosed inside, and the outer membrane of the APG fuses with a lysosome (Figure 3). This results in lysosomal degradation of the cargo contained within the APG [31]. Cargo breakdown products are recycled back into the cytosol for de novo synthesis of energy substrates, macromolecules, and organelles. Microtubule-associated protein 1A/1B light chain 3B-II (LC3-II) correlates with the number of APG present in the cell at a given time, and is a marker for monitoring autophagy.

APG can engulf cytosolic components non-specifically, or select cargo can be targeted to the APG, termed selective autophagy. Autophagy receptors and adapters impart cargo selectivity. p62 is one well-characterized autophagy receptor that binds mostly ubiquitinated proteins to bring these into the APG for subsequent autolysosomal degradation (Figure 3). p62, like LC3-II, is degraded with the cargo in the autolysosome, providing a measure for selective autophagy. Mitophagy, selective autophagic degradation of damaged and dysfunctional mitochondria, is the most fully characterized type of selective autophagy.

Regulation of autophagic activity, and the consequences for cellular health of activating or inhibiting autophagy, depend on cell type, microenvironment, and specific stimuli. In some cell types, inhibition of autophagy in response to a stressor may disrupt normal cell function and/or cause cell death, while in a different cell type, similar consequences may result from autophagy activation.

Antiretroviral drugs cause a myriad of side effects in PLWH, including hepato- and cardiotoxicity, renal dysfunction, neurotoxicity, and adipose toxicity. These deleterious effects may be due, in part, to impacts on autophagy in various cells, tissues, and/or organs. In this review, we describe what is known about how antiretroviral drugs of different classes, older ones and ones in current use, individually and in combination, and their impacts on autophagy in different cell types to contribute to clinical toxicities and comorbidities PLWH experience (Table 1). Many of the studies were performed with cell lines, and a few with primary cells or with animal models. As autophagic responses to stimuli can be different for cell lines compared to primary cells, we specify each study’s model system. We also specify the antiretroviral drug concentrations as these are relevant for comparing to in vivo serum or CSF levels. A comprehensive understanding of effects on autophagy can lead to development of antiretroviral drugs that do not impact autophagy or activate/inhibit autophagy to improve clinical outcomes in PLWH, decreasing side effects and maintaining or enhancing antiviral activity.

## 2. Reverse Transcriptase Inhibitors

Reverse transcriptase inhibitors (RTI) have been the mainstay for HIV treatment. There are two main types, nucleoside RTI (NRTI) and non-nucleoside RTI (NNRTI). A third, termed nucleoside RT translocation inhibitor (NRTTI), is under development (Figure 1 and Figure 2). RTI are associated with many side effects, including myopathy, lipodystrophy, lactic acidosis, steatohepatitis, neuropathy, neuropsychiatric changes, cutaneous reactions, and hypersensitivity reactions. These can be severe, and sometimes fatal. Newer RTI, such as tenofovir and emtricitabine, have significantly less risk for toxicity than older drugs such as zidovudine or stavudine. Several of the side/adverse effects are a result of negative impacts to mitochondria and the ER, but only a few studies examined impacts on autophagy as a possible underlying mechanism of toxicity. Mitochondrial quality control is regulated by mitophagy, and mitochondrial damage and dysfunction are major stimuli that induce autophagy/mitophagy. The findings related to effects on autophagy, including mitophagy, that RTI induce in various model systems are summarized below.

### 2.1. Efavirenz

Efavirenz (EFV) was the foundation of first-line regimens for nearly two decades. Although it is still recommended by the World Health Organization in an alternative first-line regimen for adults [32] and is used often in resource-limited settings, EFV has fallen out of favor because of its toxicities. These include neuropsychiatric effects such as sleep disturbance, decreased concentration, headache, vertigo and hallucinations, transaminitis and hepatotoxicity, cutaneous reactions, and increased risk for cardiovascular events. Mitochondrial dysfunction and ER stress, which are closely linked to autophagic processes, contribute to these side effects (Table 1). Yet, only a few studies have examined EFV effects specifically on autophagy, with various findings.

EFV can induce mitophagy. In one study, 5 and 10 µM EFV caused mitochondrial dysfunction in SH-SY5Y cells, a human neuroblastoma cell line, and concentrations above 100 nM in primary rat striatal neurons [6]. EFV also increased autophagic flux, with an increase in mitochondrial colocalization with LC3 puncta, suggesting increased mitophagy. Experiments using a tandem fluorescent mitochondria reporter confirmed EFV increased mitophagy in these cells. Mitochondrial damage was exacerbated by EFV when autophagy was pharmacologically inhibited. The authors conclude that mitophagy is a protective mechanism against EFV-induced mitochondrial toxicity in neuronal cells [6]. Autophagy plays an important role in the health and function of CNS cells, and contributes to neurodegeneration when dysregulated. While mitophagy upregulation may be considered protective, long-term upregulation from ongoing EFV exposure in PLWH may lead to autophagy exhaustion. EFV-associated neuropsychiatric side effects and HIV-associated neurocognitive disorders (HAND) are possibly attributable, in part, to dysregulated autophagy. One consideration is that there is great interindividual variability of EFV CSF levels at steady state, ranging from 6.6 to 67 nM [33,34]. There is limited information as to how much reaches brain parenchyma. The authors tested lower concentrations of EFV but found statistically significant differences only with the higher EFV concentrations. Nonetheless, further support for the role of EFV in HAND is described below in the context of EFV autophagy inhibition.

In another study, EFV effects on mitophagy were concentration-dependent. Concentrations of 25 and 50 µM EFV caused mitochondrial architectural abnormalities in Hep3B cells, a hepatocellular carcinoma cell line [7]. After 25 µM treatment, these changes corresponded to increased autophagic flux, and inhibiting autophagy exacerbated EFV effects on mitochondria, leading to apoptosis. After 50 µM treatment, however, flux was decreased despite the presence of significant mitochondrial damage, and cell death was greater after autophagy inhibition [7]. Mitochondrial colocalization with APG and lysosomes was increased in HeLa cells after EFV, suggesting that mitophagy was upregulated. The authors conclude that mitophagy upregulation after 25 µM promotes cell survival in the setting of mitochondrial damage, but that the higher EFV concentration causes mitochondrial damage exceeding the autophagic system capacity, thus inducing autophagic stress, resulting in apoptosis [7]. This same group confirmed their findings of EFV mitochondrial toxicity in a later study of Hep3B cells [8], but did not address autophagy or mitophagy with flux assays. While the daily dose of EFV usually leads to serum levels between 3.17 and 12.67 µM [35], 20–40% of people can have plasma levels far exceeding the therapeutic range, reaching as high as 30–50 µM [36,37,38], owing to major interpersonal variability of EFV pharmacokinetics. EFV toxicity to mitochondria and mitophagy likely contributes to hepatotoxicity. Notably, the defects in the neuronal cells [6] were induced at lower EFV concentrations than for hepatocytes, suggesting that different cell types have different sensitivities for responding to EFV. Autophagy in neuronal cells may be overwhelmed at lower EFV concentrations, although additional studies with CSF concentrations of EFV will be important to confirm this.

Although upregulation of autophagy is most often considered to be cytoprotective, it can also be cytotoxic. One group examined EFV effects on autophagy in normal human keratinocytes to characterize mechanisms underlying EFV-associated cutaneous reactions [9]. EFV at 10 µM triggered terminal differentiation and apoptosis that was accompanied by decreased mTOR phosphorylation, and an increase in LC3-II, beclin-1 (BECN1), and ATG5, suggesting autophagy induction. Inhibiting autophagy reversed the molecular changes and restored replication capacity of the keratinocytes. The authors conclude that EFV induction of autophagy is toxic to keratinocytes and may contribute to cutaneous reactions [9]. Underscoring the cell-type specific effect of EFV on autophagy, the same study showed that EFV caused no change in autophagy in normal human fibroblasts. Another group also suggests that autophagy induction by EFV leads to dysfunction in endothelial cells [10]. Treatment of EA.hy926 cells, an endothelial cell line, and human umbilical vein endothelial cells (HUVEC) with 32 µM EFV led to ROS production, ER stress, loss of endothelial meshwork formation, and decreased growth and viability [10]. EFV also increased APG, suggesting increased autophagy. They did not perform flux assays nor connect increased APG to the abnormal endothelial cell phenotype observed after EFV treatment [10]. It is therefore difficult to assess whether the autophagy change is a response to mitigate ROS and ER stress, or whether the change contributes to cell toxicity. It is also possible that the autophagy effect is artifact from high EFV amount that may not occur in vivo.

EFV also inhibits autophagy. In one study, treatment of hCMEC/D3 cells, a human brain endothelial cell line, and primary human brain microvascular endothelial cells (HBME) with physiologically relevant CSF concentrations of EFV resulted in significant ER stress, which most often increases autophagic flux [11]. In this model, however, flux was inhibited due to decreased association of ATG2a with ATG9, resulting in decreased PI_(3)_P and APG [11]. In another study of U251-MG cells, a human astroglioma cell line, clinically relevant serum concentrations of EFV, as opposed to those in CSF, led to abnormal mitochondrial architecture and dysfunction, and increased mitochondrial colocalization with large LC3 puncta that indicate accumulation of undigested cargo [12]. LC3-II and p62 protein were also increased. The authors conclude that autophagy is intact, but that canonical mitophagy was not activated as mitophagy adaptor protein levels were unchanged [12]. We suggest an alternative interpretation. The accumulation of autophagic proteins and large LC3 puncta, coupled with increased mitochondrial mass with no change or increased mitochondrial proteins, may represent decreased APG maturation, and therefore an inhibition of autophagic/mitophagic flux. It seems that EFV inhibited some aspect of autophagy in these glial cells; however, additional studies with CSF concentration will confirm this. Autophagy inhibition as a result of EFV in endothelial and glial cells may contribute to neuropsychiatric side effects and neurodegenerative changes that occur in many PLWH.

### 2.2. Zidovudine and Stavudine

Zidovudine (ZDV) and stavudine (d4T) together formed the backbone of many early combination ART regimens. ZDV is now less commonly used, although remains important in resource-limited settings. d4T was removed from the US market in 2020. While RTI share similar toxicity profiles, lipodystrophy syndrome and myopathy, including cardiomyopathy, are prominent with ZDV and d4T. d4T is also strongly associated with lactic acidosis, hepatomegaly with steatosis, and pancreatitis, all of which can be fatal. NRTI inhibit DNA polymerase-γ (pol-γ), the polymerase responsible for mitochondrial DNA (mtDNA) replication. This results in mtDNA depletion and mitochondrial dysfunction, and is one major mechanism by which NRTI may cause adverse effects. However, there is no correlation between NRTI-induced pol-γ inhibition and clinical toxicity, suggesting that other mechanisms, such as autophagy dysregulation, also contribute to toxicity.

ZDV and d4T appear to inhibit autophagy in most cell types examined. In two separate studies, one group characterized autophagy changes in C2C12 cells, mouse myoblasts [13], and in differentiated adipocytes from a mouse fibroblast cell line, 3T3-F442A [14], after treatment with concentrations of ZDV or d4T ranging from therapeutic maximum (C_max,_ which is approximately 6 and 3 µM, respectively) to 5× and 30× C_max_, to determine the mechanisms driving myopathy and lipodystrophy in PLWH. They show that APG maturation was significantly inhibited in both cell types dose-dependently, importantly, at C_max_ of ZDV or d4T. This was accompanied by hyperpolarization of mitochondrial membranes in the myocytes [13], accumulation of mitochondria and inhibition of lipid acquisition in the adipocytes [14], and increased ROS and decreased cell viability in both cell types [13,14]. This phenotype was recapitulated after pharmacologic and genetic inhibition of autophagy in the different cells. The authors conclude that ZDV- or d4T-mediated autophagy inhibition causes myocyte and adipocyte dysfunction, and may contribute to myopathy and lipodystrophy syndrome in PLWH. ZDV or d4T also inhibit hepatocyte autophagy, leading to dysfunction. The same group in a third study treated HepG2 and HUH7 cells, human hepatocellular carcinoma cell lines, with C_max_ of either ZDV or d4T [15]. They showed that autophagy is inhibited in conjunction with mitochondria accumulation, increased ROS, inappropriate lipid accumulation, and decreased cell viability. ZDV or d4T effects were mirrored by pharmacologic and genetic autophagy inhibition [15]. Thus, autophagy inhibition by ZDV or d4T likely contributes to steatohepatitis in PLWH. ZDV and d4T were not used in combination in these studies, but it is possible that the damage and dysregulation would be amplified when paired together, as they once were for treating PLWH.

ZDV-mediated autophagy inhibition is suggested to contribute to development of hepatocellular carcinoma. Hepatocytes from rats that had consumed ZDV had increased mTor phosphorylation and decreased ULK1 phosphorylation, suggesting inhibited autophagy induction, and increased LC3-II and p62, indicating inhibition of APG maturation [16]. Thus, ZDV inhibited both autophagy induction and degradation. Although the rats had normal serum liver biomarkers, there was decreased mtDNA, increased b-hydroxybutyrate, and increased lipid accumulation in the hepatocytes, indicating hepatocyte damage and dysfunction. The BCL-2-associated X protein/B cell lymphoma 2 (BAX/BCL2) ratio, a measure of apoptosis susceptibility, was significantly decreased after ZDV treatment, suggesting the homeostatic balance had tipped towards pro-survival, despite damage and autophagy dysregulation. The authors conclude this may favor carcinogenesis [16]. One limitation to this study is that the ZDV serum concentration was not measured. It is unknown whether the dose the rats received resulted in physiologically relevant serum ZDV concentrations.

ZDV may have a different effect on autophagy in oocytes, T cells, and endothelial cells. While mitochondrial toxicity is believed to contribute to decreased fertility in PLWH taking NRTI [39,40], one group assessed whether autophagy changes also contributed since autophagy eliminates damaged mitochondria. Oocytes from female rats who were treated with ZDV had time-dependent decreases in maturity markers, cleavage and blastocyst formation, and BCL-2, suggesting increased apoptosis [17]. There was also reduced mtDNA, and the cells produced less ATP. These defects were partially attenuated in rats treated with ZDV plus 3-MA, an autophagy inhibitor. These findings were recapitulated in additional experiments where oocytes from control rats were treated ex vivo with 40 µM ZDV ± 3-MA. The authors additionally found increased ATG5, ATG7, BCN1 mRNA, and increased LC3-II protein, which could be partially reversed by 3-MA. They conclude that NRTI contributes to low fertility by causing oocyte dysfunction that is mediated, in part, by autophagy [17]. While the rats received human-referenced ZDV doses, serum drug levels were not tested to confirm therapeutic ZDV levels. Oocytes were also treated ex vivo with a high amount of ZDV. Increased autophagy may contribute to oocyte dysfunction. Additional flux assays with lower ZDV amounts will confirm the conclusion.

PLWH taking ZDV-based regimens were had impaired immunologic recovery relative to those taking non ZDV-based regimens. Since ZDV inhibits autophagy in other cell types, the authors of one study hypothesized that inhibition of autophagy in T cells by ZDV may lead to poor T cell survival, resulting in suboptimal T cell recovery in ZDV-treated PLWH [18]. Primary T cells were isolated from healthy donors and treated with 5 µM ZDV. There was an increase in the percent of CD4+ and CD8+ T cells expressing mitochondrial specific ROS (mitoROS), and cells were more susceptible to apoptosis. They also showed an increased percentage of CD4+ T cells with mitoROS from PLWH on ZDV-based regimens as compared to PLWH not taking a ZDV-based regimen. Endogenous LC3, measured by flow cytometry, in ZDV-treated T cells from healthy donors did not differ from the two groups of PLWH with differently based ART regimens. While healthy donor T cells were treated with ZDV for only 6 h, which may be too short exposure time to induce autophagy, the minimum duration of treatment of PLWH with an ZDV-based regimen was 1 year. They conclude mitophagy was not impacted by ZDV [18]. Additional flux studies would confirm their findings.

In another study, ZDV is suggested to induce mitophagy in human umbilical vein endothelial cells (HUVEC), contributing to cardiovascular complications in PLWH [19]. Treatment with peak steady state plasma concentration of ZDV led to multiple defects in the electron transport chain, resulting in decreased ATP production and increased ROS. There was an increase in Nix, a selective autophagy receptor that transports mitochondria to APG. Mitochondria also colocalized with lysosomes more frequently after ZDV, and the LC3-II/I ratio was increased, suggesting an upregulation of mitophagy in response to ZDV. Although the LC3-II/I ratio is used commonly to assess autophagic flux, as there is increased conversion of LC3-I to LC3-II when autophagy is induced, use of this ratio is controversial [41].

### 2.3. Lamivudine

Lamivudine (3TC) remains an important component of the antiretroviral arsenal, and can also be used for post-exposure prophylaxis to prevent HIV infection after potential exposure [42]. 3TC was used in the same studies of myocytes, adipocytes, and HepG2 and HUH7 hepatocytes described above for ZDV and d4T [13,14,15]. 3TC had no effect on mitochondria nor on autophagy in any of the cell types [13,14,15]. However, the peak steady state amount of 3TC, which is approximately 8 µM, caused significant electron transport chain dysfunction in HUVEC with increased ROS and decreased ATP production, and increased the LC3-II/I ratio as well as Nix, suggesting increased mitophagy [19]. The authors conclude 3TC may contribute to endothelial cell injury and, thus, cardiovascular complications in PLWH as a result of induction of mitophagy. Rat oocyte dysfunction also appeared to be mediated, in part, by autophagy induction as a result of 30 µM 3TC, as described above for ZDV [17], although this concentration is higher than peak steady state. While 3TC may not cause as much clinical toxicity as ZDV, it may induce autophagy in a cell-specific manner, contributing to endothelial cell and oocyte dysfunction, and cardiovascular complications and low fertility, respectively.

## 3. Protease Inhibitors

Protease inhibitors (PI) were introduced into ART regimens eight years after the first RTI (Figure 1 and Figure 2) and remain an important component of modern ART regimens. PI can cause significant metabolic toxicity including insulin resistance, lipodystrophy, dyslipidemia, hepatoxicity, and nephrotoxicity. Many molecular mechanisms contribute to these toxicities. These include inhibiting insulin-mediated glucose uptake by downregulating glucose transporter-4, GLUT4, and inhibiting proteasomal degradation of lipid binding proteins, including apolipoprotein B, ApoB. Given its significant role in proteostasis and metabolic stress response, autophagy may also be central to the toxic effects PI have on cell types, leading to side effects. There are few studies that examined PI effects on autophagy as they relate to toxicity. These are summarized below.

### 3.1. Lopinavir/Ritonavir

Ritonavir (RTV) was initially used for its antiretroviral effect, but it strongly inhibits CYP3A4 liver enzyme, thereby increasing levels of other PI. It is used now strictly for its PI-boosting activity. Lopinavir (LPV) is unique in that it was not formulated for use independently from ritonavir.

One group studied the effects of different concentrations of LPV ± RTV on mouse 3T3-L1 matured adipocytes and on human SGBS matured adipocytes [20]. Concentrations of 12.5 and 25 µM LPV induced ER stress and apoptosis that was greater after the same concentrations of LPV + RTV. The minimum serum therapeutic concentration (C_min_) of LPV is approximately 4.5 µM and the C_max_ is 21 µM when dosed twice a day with RTV and without other antiretroviral drugs [43]. LPV/RTV increased the number of APG as shown by autophagic flux assay as well as fluorescent and electron microscopy. p62 was also increased. These results suggest that despite causing ER stress, LPV/RTV inhibits autophagic flux in adipocytes, potentially contributing to lipodystrophy and dyslipidemia in PLWH [20].

Another study examined LPV/RTV-induced hepatoxicity and autophagy [21]. Primary mouse hepatocytes, Kupffer cells, and hepatocellular stellate cells were treated with approximately 36 µM LPV/RTV, which led to increased binding immunoglobulin protein (BIP) and C/EBP homologous protein (CHOP), markers of ER stress. The nuclear factor erythroid 2-related factor 2 (NRF2)-mediated antioxidant response was also inhibited. ER stress and oxidants can induce autophagy. They found increased LC3-II after LPV/RTV, concluding autophagy was induced [21]. Confirmation of autophagy induction in these cells with a lower amount of LPV/RTV would further support the cell specific effects of antiretroviral drugs on autophagy.

PI effects on autophagy in placental cells may disrupt normal placental development and function. PI use during pregnancy has been variably associated with premature delivery, low birth weight, small for gestational age, maternal and neonatal endocrine dysfunction, and neonatal hematologic abnormalities [44,45,46,47,48,49] as well as abnormal placental morphology, cord insertion, and vasculature [48,50,51,52]. ER stress underlies many toxicities associated with PI, and ER stress is important to the physiology and pathophysiology of the placenta [53]. There is one study that assessed ER stress and autophagy associated with LPV/RTV to identify possible placenta damaging effects of PI [22]. 3A-subE and JEG-3 cells, immortalized human placental and human choriocarcinoma cell lines, respectively, were treated with 12.7 µM LPV + 2.77 µM RTV, which corresponds to a fixed dose tablet of 200 mg/50 mg, and ER stress was assessed by presence of unconventionally spliced X-box binding protein 1 (XBP1) mRNA. XBP1, a major transcription factor that upregulates ER stress response genes, is activated in the cytosol when spliced at an unconventional splice site. The presence of this unconventionally spliced XBP1 indicates ER stress. There was significantly more unconventionally spliced XBP1 in each cell type after LPV/RTV. More APG was also found in these same cells. They also compared spliced XBP1 in placenta of women without HIV and of women with HIV who were taking antiretroviral drugs. There was increased spliced XBP1 from the women with HIV on ART, and while there was significant inter-individual variability in spliced XBP1, the placentas with the highest levels of spliced XBP1 were from women who were taking regimens that contained LPV/RTV or atazanavir, another PI. They conclude that ER stress-induced by LPV/RTV causes autophagy upregulation [22]. Future autophagy-directed studies of LPV/RTV-treated 3A-subE and/or JEG-3 cells would be helpful to distinguish autophagy induction from inhibition that led to the increased APG.

### 3.2. Atazanavir

Atazanavir (ATV) is one of the preferred first-line medications for pregnant women [32]. It appears to have less impacts on lipids, appearing less likely to cause lipodystrophy. It may also induce autophagy. In the study described above of LPV/RTV effects on the placenta, one of the highest placenta levels of spliced XBP1 was from a woman taking an ART regimen containing ATV [22]. Similar to LPV/RTV treatment, JEG-3 cells treated with ATV had increased numbers of APG [22], although this effect was seen at 12.5 µM ATV and higher. This is more than the reported serum C_max_ of approximately 7 µM in pregnant women [22,54].

Another study provides additional evidence to suggest ATV does induce autophagy. To determine whether autophagy changes in adipocytes resulting from ATV contributes to lipodystrophy, SW872 cells, a human pre-adipocyte cell line, were treated with concentrations ranging 10 to 200 µM ATV and autophagy was assessed [23]. Cells had increased numbers of APG by light and electron microscopy and increased LC3-II by Western blotting after 50 µM. These effects were similar to those of serum-starvation, a classic autophagy inducer. Mitochondrial dysfunction was present at low ATV doses, and there was a dose response with increasing dysfunction at higher concentrations of ATV and significant apoptosis. The authors conclude that ATV activates autophagy/mitophagy and may contribute to lipodystrophy [23]. The mean C_max_ plus the standard deviation of ritonavir-boosted ATV serum is approximately 12 µM in subjects with HIV [55], although there is great inter- and intra-individual variability in drug concentrations regardless of whether ATV is boosted or not [56,57,58]. Another limitation to the study is that autophagy was not correlated with cell differentiation, lipid content, or apoptosis. Additionally, increased mitochondrial content would not be expected unless mitophagy were induced to such a degree that “autophagic stress” occurred, as was the case for EFV treatment of Hep3b cells discussed above [7].

### 3.3. Saquinavir

Saquinavir (SQV) has played a critical role in the development of combination regimens (Figure 1). To characterize lipodystrophy, one group examined the effects of 30 µM SQV on Chub-S7 cells, transformed human adipocytes [24]. This amount was chosen for its known cytotoxicity [59]. Ritonavir-boosted SQV serum C_max_ ranges from 2.1 to 26 µM [60]. SQV led to lipid loss and downregulation of genes associated with differentiation in addition to proteosome inhibition and ER stress. They also found that SQV increased the expression of genes related to autophagy and caused an increase in APG numbers. They conclude that SQV may inhibit the proteosome, thus inducing the unfolded protein response resulting in autophagic breakdown of lipid deposits [24]. Flux assays would provide additional confirmation of their studies.

## 4. Combination Antiretroviral Drugs

While monotherapy showed promising initial results, it had only limited success [61,62]. HIV mutates rapidly under the selection pressure of just one drug, developing drug resistance easily. The need for combination therapy was recognized within just a few short years after introduction of ZDV [63], and the first combination pill, Combivir^®^, was approved in 1997 (Figure 1). Combination therapy truly transformed HIV from a fatal disease to a manageable chronic infection. Current guidelines advise the use of a three-drug regimen consisting of two NRTIs and either a protease inhibitor or integrase inhibitor [32]. Two-drug regimens consisting of one NRTI and one integrase inhibitor are also used [32]. There are relatively few studies of ART combinations on autophagy, and all have been published in the last five years. The long-term impacts of combination regimens on autophagy are important to identify so that regimens can be optimized both to reduce viral load and to minimize side effects.

Three comprehensive studies showed that three-drug regimens containing two NRTI plus an ISTI inhibit autophagy, and may contribute to HAND in PLWH [25,26,27]. A two-part study addressed the effects of tenofovir disoproxil fumarate (TDF), emtricitabine (FTC), and dolutegravir (DTG) on autophagy and inflammatory genes/proteins in primary rat microglia. After 24 h treatment with TDF + FTC + DTG at 5 µM for each, lysosome quantity was decreased, pH was increased, and the amount of mature cathepsin D—a lysosomal protease—was decreased. Autophagic flux assays showed this abnormal lysosome function contributed to inhibited APG maturation [25]. By dual fluorescent reporter, there were also more total red puncta compared to untreated control, but similar in appearance to rapamycin, indicating more total autophagic vesicles. Therefore, the ART cocktail appears to induce autophagy yet impair maturation, leading to decreased flux. These changes correlated with an increase in mRNA for many inflammatory cytokines, although protein levels were not measured. They conclude that ART-induced dysregulated autophagy contributes to increased neuroinflammation, thus contributing to HAND pathogenesis [25]. The authors followed up this study by evaluating the role of ROS in the autophagy/lysosome dysfunction in microglia caused by 5 µM each of TDF + FTC + DTG [26]. Treatment of primary rat microglia with this cocktail increased ROS, and pre-treatment with the ROS scavenger, N-acetylcysteine (NAC), neutralized the inhibitory effect of these drugs on autophagy. This correlated with a return to baseline levels of mRNA for *IL-1β*, *IL-6*, and *TNF-α*, suggesting that ROS impacted both inflammatory gene expression and inhibited autophagic flux in rat microglia [26]. The authors then examined the effects of NAC on the brains of TDF + FTC + DTG treated HIV Tg rats. The rats were injected intraperitoneally with doses of drugs that were approximately 6× higher than human drug doses, to account for increased metabolic rate of rats [26]. Recapitulating in vitro results, TDF + FTC + DTG diminished total lysosome activity in the brain, accompanied by increased p62 and LC3II as detected by Western blot. This correlated with higher levels of total IL-1β protein and, overall, more microglia in the brain. Treatment with TDF + FTC + DTG also decreased microglial process length, indicating microglial activation. NAC pre-treatment reversed this, suggesting a role for oxidative stress in these processes in vivo [26]. These studies show clear autophagy inhibition, although mean CSF C_max_ for TDF is 9.6 nM, 440 nM for FTC, and between 34.6 and 74 nM for DTG [64,65]. The mechanisms by which oxidative stress causes lysosome impairment remain to be characterized.

The third study, from our laboratory, demonstrated that a combination of tenofovir (TEN) + FTC + RAL inhibits autophagy in primary human astrocytes at concentrations consistent with CSF levels [27,64,65]. Using several techniques, including two different flux assays, we showed that APG biogenesis was inhibited after 24 h as well as after 7 days of daily treatment with the cocktail. We also showed increased p62 protein and decreased p62 flux without an increase in p62 transcription at multiple time points, suggesting that p62-mediated selective autophagy is inhibited [27]. The mechanisms that mediate decreased selective autophagy may be related to inhibited APG biogenesis, which decreases the amount of p62 available for degradation by autophagy. Future studies will address the mechanisms that mediate autophagy inhibition in primary human astrocytes, and how autophagy dysfunction in astrocytes contributes to HAND in PLWH.

There is one study that suggests another combination of antiretroviral drugs inhibits autophagy [28]. The effects of 24 h and 7 days of 0.1 to 10 µM ZDV, 0.1 to 1 µM nevirapine (NPV), 5 to 50 nM SQV, and 2 o 20 µM 118-D-24, an unnamed integrase inhibitor (IntInh), on primary rat cerebrocortical neurons were examined. These concentrations were chosen for reported ranges of therapeutic serum doses for each drug [28]. ATP generation was decreased after both 24 h and 7 days of treatment with this four-drug cocktail. Immunofluorescence after 7 days, but not 24 h, of treatment showed decreased MAP-2 and SYP, markers of neuronal health. LC3-II protein was increased, p62 protein decreased, and LC3 puncta were also increased, suggesting this antiretroviral drug combination induced autophagy [28]. Rapamycin could recover ATP production after 24 h of ART, but failed to do so after 7 days of ART. This suggests that while ART may induce autophagy, there may also be a maturation defect since rapamycin could recover the ART-induced decrease in ATP. Flux analyses were not performed to rule out impaired maturation. That rapamycin failed to restore ATP generation after 7 days of ART indicates that longer term exposure to ART may impair the ability of neurons to respond appropriately to autophagy inducers, at least when the inducer mediates its autophagy effect through mTOR inhibition. Alternatively, the link between rapamycin and ATP production may not be mediated through autophagy. The mechanisms by which this ART mediates these changes remain to be determined. It is important to note that these drugs were not used at CSF concentrations, and are not used in this specific combination to treat HIV.

The authors of another study of HAND in the context of opioid use also suggest that combination ART is inhibitory to autophagy [29]. They hypothesized that opioids reduce the antiviral effects of ART in astrocytes infected with HIV in vitro, and that this effect relates to changes in autophagy. Treatment of infected astrocytes for 7 days with morphine plus 10 µM each of FTC + RTV + ATV (ERA) increased viral output and inflammatory cytokine secretion relative to ERA alone, suggesting that opioid intake may counteract antiretroviral activity and the beneficial impact of ART on inflammation in astrocytes. This effect on virus and cytokines correlated with an increase in p62 gene transcription and protein [29]. While there was no change in LC3 protein level, it is unclear whether LC3-I, LC3-II, or both were quantified together, nor were flux assays performed. Thus, effects on autophagy cannot determined. p62 is a gene target of the Kelch-like ECH-associated protein 1 (KEAP1)/NRF2 antioxidant response system. It is possible that increased p62 transcription and protein levels may be a result of oxidative stress. There was evidence of compromised mitochondrial membrane integrity; however, ERA nor morphine + ERA did not significantly increase ROS [29]. The same studies were performed with a different combination of drugs that included 10 µM each of LPV+ abacavir (ABC) and RAL (LAR). The effects on viral and cytokines, as well as on p62, were not found after treatment with morphine + LAR [29]. These suggest an effect unique to a specific combination of antiretroviral drugs, thus highlighting the importance of performing studies with contemporary and approved ART regimens to assess their potential toxicity in PLWH, as different combinations may induce different defects. Additional studies with CSF concentrations of drugs will confirm their conclusion. Another limitation to their study is that whether astrocytes are productively infected in the brains of PLWH in the ART era remains controversial [66].

To characterize further immune senescence and abnormal chronic inflammation in PLWH, one group compared gene expression of cytogaligin (*GALIG*), a pro-apoptotic gene, induced myeloid leukemia cell differentiation protein (*MCL1*), an anti-apoptotic gene, and seven autophagy-related genes in peripheral blood mononuclear cells (PBMC) from 27 PLWH on various ART regimens to those from people uninfected with HIV [30]. The median duration of ART was 11 years, and the median undetectable viral load duration was 8.4 years. PBMC from PLWH had increased gene expression of both *GALIG* and *MCL1* compared to uninfected individuals. PBMC from PLWH also had decreased *BECN1* and increased expression of two of four ATG8 family member genes, *LC3* and gamma-aminobutyric acid receptor-associated protein-like 2 (*GABARAPL2*). While there was no difference in *GABARAPL1* or in *ATG9A* mRNA, they found that *GABARAPL1* and *ATG9A* levels were correlated, and machine learning analyses of *GABARAPL1* plus *ATG9A* was able to classify someone as having HIV or not with 94.5% predictive power. The authors conclude that these gene expression changes may contribute to immune senescence and chronic inflammation in PLWH [30]. The specific drugs in the ART regimens were not detailed, so the contributions of specific combinations to the observed changes are not known. Characterization of transcriptional regulation of autophagic activity is still ongoing [41,67]. Thus, what these data may signify in terms of changes in autophagy in PBMC from PLWH on ART is not well understood, and future studies related to autophagy proteins and flux will be important to increase our understanding of the role of ART and autophagy in immune senescence and chronic inflammation.

The study discussed above relating EFV to autophagy inhibition in human brain endothelial cells first examined ER stress and autophagy after treatment with either of two ART cocktails, EFV + TEN + FTC (cART1) or ATV/RTV + TEN + FTC (cART2) [11]. Concentrations for each drug reflected in vivo serum levels. While both cocktails induced ER stress, cART1, which contained EFV, did so to a greater degree. It was cART1, and not cART2, that decreased total LC3-II as well as the LC3-II/I ratio. They then tested the effects of the individual drugs in cART1 on the ER and autophagy, which led them to determine that it was EFV specifically that mediates the dysfunctional phenotype [11]. Their data support drug-specific effects on autophagy, and highlight the importance of determining effects of individual drugs as well as drugs in combination as they relate to changes in autophagy.

## 5. Conclusions

The lives of PLWH are dramatically prolonged due to highly efficacious antiretroviral therapy. As antiretroviral drugs have evolved into simpler regimens with improved toxicity profiles, PLWH have experienced significant improvement in the quality of their lives as well. Despite the great advances in treating HIV, antiretroviral drugs are incapable of eliminating it from the body, thus necessitating life-long treatment. For this reason, long-term side effects and toxicities for PLWH remain a significant concern.

Autophagy is vital for cell homeostasis for mitigating stress, performing quality control, and regulating many cell processes. While mitochondrial dysfunction and ER stress play an important role in antiretroviral toxicity, it is evident from the studies reviewed here that changes in autophagy also play a role in mediating ART toxicity and side effects. While direct or indirect effects on mitochondria and ER may cause some of the autophagy changes described, ART also appears to impact autophagy independently of mitochondria dysfunction and ER stress. Cells respond to mitochondrial or ER stress by inducing autophagy. However, as highlighted in these reviewed studies, autophagy upregulation can also lead to autophagy exhaustion [7], may be toxic by itself [17,18,27], or autophagy may fail to be upregulated or is inhibited [11,12,13,14,15,20,25,26,27], subsequently leading to decreased cell viability and death (Table 1). Thus, these studies reinforce the major significance of autophagy dysregulation as a mechanism underlying drug toxicity.

These studies also underscore that antiretroviral drugs impact autophagy in different ways, and that effects are specific to each drug, and to each cell type (Table 1). Some of the studies reviewed here tested multiple antiretroviral drugs individually, including relatively newer ones [12,19,21,22,29]. We reviewed drugs with positive findings, but there were drugs that did not appear to have an effect. For example, darunavir, RAL, and rilpivirine (Figure 1), did not appear to impact autophagy in Hep3B cells or primary rate neurons [8]. Etravirine (Figure 1) did not appear to impact autophagy in brain endothelial cells from HIV transgenic mice [11], nor did TEN in HUVEC [19]. It is interesting to note that EFV has perhaps the most ascribed side effects, and seems to be the drug that impacts autophagy in more ways than other antiretroviral drugs. It is also the most studied antiretroviral drug in the context of autophagy, so it could be that other drugs have just as much variability in their impact on this process, but that fewer studies related to autophagy have been performed with other drugs. For example, two studies examined ER toxicity or apoptosis with multiple individual drugs and pursued autophagy experiments only with the drugs that caused significant ER stress or cell death [20,23]. As autophagy changes can occur independent of mitochondrial or ER stress, and autophagy dysfunction itself is cytotoxic, it will be important to perform autophagy directed studies with these drugs as well. RAL is the only ISTI that has been studied independently from a drug combination (Table 1) [8]. To our best knowledge, there are no studies of the fusion inhibitor nor entry inhibitors on this topic.

It is important to note that many studies did not perform flux assays, several different autophagy assays, or link autophagy changes to the toxicity/side effect of interest. Therefore, our knowledge regarding effects of individual or combination drugs on autophagy is incomplete. Ideally, multiple different assays best suited for the model system should be used, and interpretation of data is performed within a framework of established criteria for determining autophagy changes [41]. This applies to all autophagy-related studies, including those examining selective autophagy, microautophagy, and chaperone-mediated autophagy. This would enable more accurate assessment of whether a specific antiretroviral drug or ART cocktail truly impacts autophagy, which step(s) are affected, and how the impacts contribute to cell toxicity. Additional research is needed to more completely characterize the total effects of antiretroviral drugs on autophagy in specific cell types as they relate to toxicity. This is relevant for antiretroviral drug development as understanding the impacts on autophagy could guide drug design to mitigate impacts on autophagy to decrease toxicity. In addition, an individual’s ART regimen could be selected with consideration of effects on autophagy: one drug’s inhibitory effect on autophagy could counterbalance another drug’s inducing effect. In addition, declining autophagic activity is associated with normal aging [68], and PLWH develop age-related comorbidities prematurely [69,70,71]. Antiretroviral effects on autophagy may be contributing to this premature aging. ART regimens could be selected to have less impact on autophagy to decrease or slow this premature aging.

Additional limitations of some studies are related to cell types, and to antiretroviral concentrations. Many studies were performed with transformed or immortalized cell lines, or lines different from the organ of interest. While cell lines offer many advantages over primary cells, autophagic responses to stimuli in lines can be different from those in primary cells. It is important to pursue additional studies in primary cells to more closely approximate the in vivo state. In addition, antiretroviral concentrations used in some studies did not correspond with known human plasma or CSF concentrations. While some drugs have large inter- and intra-individual concentration variability, for example, EFV and ATV, effects on autophagy with high in vitro drug concentrations may result in misleading findings. It has been posited that drug concentrations in tissue are potentially higher than in serum, perhaps with the exception of the brain, due to the blood–brain barrier and low or poor CNS tissue penetration of many antiretroviral drugs [61,72,73]. Nonetheless, use of concentrations reflective of serum or CSF levels, depending on the tissue of interest, will more closely replicate the in vivo experience and minimize identification of artifact. However, in animal studies, concentrations may need to be adjusted to account for their higher rates of metabolism. Lastly, exposure time and dosing interval of antiretroviral drugs in experiments should be considered as, for the most part, PLWH are taking life-long medications daily. These are important for advancing our knowledge of the impact of antiretroviral drugs on autophagy as they relate to clinical side effects and toxicities.

Many studies reviewed here examined older drugs that have limited or no use in the treatment of HIV in the current ART era. Nevertheless, understanding antiretroviral drug effects on autophagy remains highly relevant as there is substantial and growing interest in modulating autophagy for therapeutic purposes. Several antiretroviral drugs are being repurposed for the treatment of other diseases due to their impacts on autophagy. EFV, nelfinavir (a PI), zalcitabine (an RTI), and others [72,73,74,75,76,77,78] have been examined as potential adjunct chemotherapeutic agents as their effects on autophagy are toxic to certain cancer cell types. D4T has been examined as potential therapy for Alzheimer’s disease as it appears to increase macrophage Aβ phagocytosis, an effect that may be mediated, in part, by autophagy [79]. Understanding effects on autophagy provides opportunity to maximize treatment options in the cancer setting, and in other arenas where treatment options are limited, such as in neurodegenerative conditions.

Autophagy could also be harnessed to effect improvements in treatment of HIV. This is being examined for long-acting antiretroviral drug nanoformulations. These offer new opportunities for overcoming adherence challenges that PLWH may face [80]. The first long-acting injectable, cabotegravir, an ISTI, was approved by the FDA in early 2021, and is dosed just monthly along with a long-acting injectable formulation of rilpivirine, an NNRTI (Figure 1). They also offer opportunities to improve therapeutic outcomes by facilitating controlled drug release, extending drug half-lives, and potentially increasing penetrance into viral reservoirs. This would decrease viral mutation rates, decrease reservoir burden, and also potentially decrease toxicity as longer half-lives could translate to lower therapeutic doses [80]. One group is studying compounds that increase autophagy to enhance longevity and slow release of macrophage antiretroviral drug depots, which they have shown can improve antiretroviral responses [81,82]. Understanding the crosstalk between autophagy and antiretroviral drugs has the potential to facilitate development of antiretroviral drugs with less toxicity, but also to improve antiretroviral efficacy.

In summary, autophagy dysfunction mediates antiretroviral toxicity, and effects are cell-type- and also drug-specific. However, ART-induced autophagy dysfunction remains incompletely understood, and more studies are needed. This knowledge could drive improvements in antiretroviral drug molecules, that have reduced and/or beneficial effects on autophagy, to continue improving lives for PLWH as well as possibly sufferers of other diseases such as cancer and neurodegeneration.

## Data Availability

Not applicable.
